# P465L‐PPARγ mutation confers partial resistance to the hypolipidaemic action of fibrates

**DOI:** 10.1111/dom.13370

**Published:** 2018-06-27

**Authors:** Sergio Rodriguez‐Cuenca, Stefania Carobbio, Gwendolyn Barceló‐Coblijn, Xavier Prieur, Joana Relat, Ramon Amat, Mark Campbell, Ana Rita Dias, Myriam Bahri, Sarah L. Gray, Antonio Vidal‐Puig

**Affiliations:** ^1^ University of Cambridge Metabolic Research Laboratories, Level 4 Wellcome Trust‐MRC Institute of Metabolic Science Cambridge UK; ^2^ Wellcome Trust Sanger Institute, Wellcome Trust Genome Campus Hinxton UK; ^3^ Institut d'Investigació Sanitària Illes Balears (IdISBa, Balearic Islands Health Research Institute) Palma Spain; ^4^ Département des Sciences de la Vie, L'Institut du Thorax, INSERM, CNRS Université de Nantes Nantes France; ^5^ Department of Nutrition, Food Science and Gastronomy, School of Pharmacy and Food Science, Food and Nutrition Torribera Campus. University of Barcelona (UB), Santa Coloma de Gramenet (Spain); INSA‐UB, Nutrition and Food Safety Research Institute University of Barcelona Barcelona Spain; ^6^ Cell Signaling Unit, Departament de Ciències Experimentals i de la Salut Universitat Pompeu Fabra (UPF) Barcelona Spain; ^7^ Northern Medical Program University of Northern British Columbia Prince George Canada

**Keywords:** fatty liver, fibrates, lipodystrophy, P465L‐PPARγ, PPARα

## Abstract

**Aims:**

Familial partial lipodystrophic syndrome 3 (FPLD3) is associated with mutations in the transcription factor PPARγ. One of these mutations, the P467L, confers a dominant negative effect. We and others have previously investigated the pathophysiology associated with this mutation using a humanized mouse model that recapitulates most of the clinical symptoms observed in patients who have been phenotyped under different experimental conditions. One of the key clinical manifestations observed, both in humans and mouse models, is the ectopic accumulation of fat in the liver. With this study we aim to dissect the molecular mechanisms that contribute to the excessive accumulation of lipids in the liver and characterize the negative effect of this PPARγ mutation on the activity of PPARα *in vivo* when activated by fibrates.

**Material and Methods:**

P465L‐PPAR mutant and wild‐type mice were divided into 8 experimental groups, 4 different conditions per genotype. Briefly, mice were fed a chow diet or a high‐fat diet (HFD 45% Kcal from fat) for a period of 28 days and treated with WY14643 or vehicle for five days before culling. At the end of the experiment, tissues and plasma were collected. We performed extensive gene expression, fatty acid composition and histological analysis in the livers. The serum collected was used to measure several metabolites and to perform basic lipoprotein profile.

**Results:**

P465L mice showed increased levels of insulin and free fatty acids (FFA) as well as increased liver steatosis. They also exhibit decreased levels of very low density lipoproteins (VLDL) when fed an HFD. We also provide evidence of impaired expression of a number of well‐established PPARα target genes in the P465L mutant livers.

**Conclusion:**

Our data demonstrate that P465L confers partial resistance to the hypolipidemic action of fibrates. These results show that the fatty liver phenotype observed in P465L mutant mice is not only the consequence of dysfunctional adipose tissue, but also involves defective liver metabolism. All in all, the deleterious effects of P465L‐PPARγ mutation may be magnified by their collateral negative effect on PPARα function.

## 1 INTRODUCTION

Peroxisome proliferator‐activated receptors (PPARs) are nuclear receptors that regulate energy homeostasis and coordinate biochemical processes that are involved in anabolic and catabolic processes. This family of transcription factors comprises 3 members: PPARγ (with 2 isoforms: PPARγ1 and PPARγ2), PPARα and PPARδ.^1^

PPARγ regulates adipose tissue development and expansion, and harmonizes the functional balance between lipogenic and lipolytic programmes.^2^ Genetic defects in PPARγ cause severe metabolic lipodystrophy phenotypes,^3^ known as familial partial lipodystrophy syndrome type III (FPLD3).^4^ Among patients suffering from this syndrome, those carrying the P467L mutation (rs121909244) exhibit a lipodystrophic phenotype, hypertension, hyperglycaemia, hepatic steatosis, and severe dyslipidaemia, a complex phenotype that is partially recapitulated in the humanized P465L‐PPARγ mutant mouse under different nutritional and genetically induced challenges, such as high‐fat diet (HFD) feeding, and when backcrossed into ob/ob, apoliporotein E knockout (APOEKO) and Akita murine genetic backgrounds.^5^, ^6^, ^7^ The isoform PPARγ2 is, under physiological conditions, preferentially expressed in WAT and BAT. Other organs, such as the liver, predominantly express the PPARγ1 isoform, but can, under pathological conditions such as overnutrition and obesity, induce *de novo* the expression of the PPARγ2 isoform.^8^ This indicates that the types and relative amounts of PPARs coexisting in the same cell/tissue under specific physiological and pathophysiological conditions vary according to specific nutritional status and metabolic adaptations.

PPARα is another important member of the PPAR family, which plays a fundamental role in lipid oxidation and biosynthesis, gluconeogenesis, cholesterol catabolism and ketogenesis.^9^ PPARα is detected in tissues characterized by high rates of β‐oxidation, such as heart, skeletal muscle and liver.^10^ Conversely, genetic ablation of PPARα in mice has confirmed its preferential involvement in fatty acid oxidation and has confirmed that, when PPARα is dysfunctional, it causes hepatic steatosis^11^ and severe fasting hypoglycaemia.^12^ According to the role of PPARα in controlling fatty acid metabolism, fibrates, a class of synthetic PPARα ligands, exert beneficial metabolic effects in patients with metabolic syndrome and in rodent models of obesity, insulin resistance and diabetes. For instance, a well‐established fibrate, WY14643 (pirinixic acid), decreases plasma triglycerides, reduces adiposity and improves hepatic steatosis and insulin sensitivity in lipoatrophic mice.^13^

Given that PPARs share common co‐activators, co‐repressors and partners, as well as DNA responsive elements (PPRE), we hypothesized that the changes in expression patterns or activity of PPARs may affect the transactivation capacity of the members of the family. *In vitro* evidence indicates that PPARs exhibit promiscuity in their binding to specific coactivators/corepressors, known to be involved in complex functional crosstalks. This has been partially addressed *in vitro* by showing that mutants for PPARα, such as a dominant negative^14^ or a ligand‐binding domain‐lacking mutant,^15^ exert cross‐inhibition of the wild type (WT) form of PPARα, and also of PPARγ and PPARδ, by competition for coactivators.^14^ Similarly, several PPARγ dominant negative mutants have been shown to repress the activity of PPARα *in vitro*.^14^, ^16^

The P467L‐PPARγ mutation exerts a dominant negative effect on WT PPARγ *in vitro* by reducing the promoter turnover rate, by out‐competing the WT receptor for promoter binding sites, and by attenuating the release of a corepressor and recruitment coactivator.^17^, ^18^, ^19^, ^20^ The pathophysiological relevance of this crosstalk between PPARs *in vivo* has not been studied.

Our laboratory has previously shown that the humanized murine model P465L‐PPARγ developed hepatic steatosis after 4 months on an HFD^5^ in the same way that P467L human carriers do.^21^ Here, we dissect the mechanisms leading to this phenotype *in vivo* and propose that the P465L‐PPARγ mutation increases susceptibility to fatty liver through a mechanism that involves a partial deficiency of the transactivation capacity of PPARα. We found that P465L mice have increased levels of insulin and FFA, both risk factors associated with fatty liver. P465L mice also display decreased levels of VLDL when fed an HFD and a partially impaired response to the hypolipidaemic action of WY14643. Moreover, gene expression profiling revealed that P465L‐PPARγ negatively impacts on the transcriptional activation and repression mediated by PPARα agonists in a variety of metabolic pathways.

## 2 MATERIALS AND METHODS

### 2.1 Animals

P465L/+ PPARγ mice were generated as described previously.^5^ All animals used in this study were fed a standard chow diet or an HFD (45% Kcal from fat) *ad libitum* and were housed at 24°C with a 12‐hour light cycle. The mice were divided into groups of 7‐8. For both genotypes, mice were distributed into the following groups: chow diet/vehicle, chow diet/WY14643, HFD/vehicle and HFD/WY14643. The HFD was initiated when mice were 5 weeks old, for a period of 28 days. Mice were then injected intraperitoneally with vehicle or WY14643 (25 mg/kg/day) for 5 days. Additionally, we used WT and P465L mice on a chow diet and an HFD (45% Kcal from fat) for a period of 12 weeks. We culled them in fed and fasting conditions (overnight).

This research was regulated under the Animals (Scientific Procedures) Act of 1986, Amendment Regulations 2012, following ethical review by the University of Cambridge, Animal Welfare and Ethical Review Body (AWERB).

### 2.2 Blood biochemistry

Enzymatic assay kits were used for determination of plasma‐free fatty acids (Roche), triglycerides (Siemens), glucose and insulin (Meso Scale Diagnostics, Rockville, Maryland) according to manufacturers' instructions.

### 2.3 Liver triglycerides content

Hepatic lipid content was measured using the Folch method as described previously.^22^

### 2.4 Lipoprotein separation by fast protein liquid chromatography (FPLC)

Pooled plasma of each experimental group was used for the isolation of lipoproteins using FPLC according to the protocol of the Diabetic Complications Consortium (https://www.diacomp.org/shared/document.aspx?id=14&docType=Protocol).

### 2.5 Glycogen determination

Hepatic glycogen content was measured as described previously.^23^

### 2.6 Histology

Livers were dissected and fixed in 10% formalin; they were then cryoprotected (20% sucrose) and frozen in chilled isopentane prior to sectioning using a cryostat. Cryosectioned tissue was stained for lipid with Oil Red O (Sigma‐Aldrich, St. Louis, Missouri).

### 2.7 Lipid analysis

Liver tissue was homogenized with a tissue blender. Lipids were extracted using n‐hexane/2‐propanol. Tissue extracts were centrifuged at 1000 *g* to pellet debris. The lipid‐containing organic phase was decanted and stored under nitrogen at −80 °C until analysis.^24^, ^25^ Total lipids were subjected to base catalyzed transesterification, converting the acyl chains to fatty acid methyl esters (FAME).^26^ Heptadecanoic acid (17:0) was used as the internal standard. Individual FAMEs were separated by gas liquid chromatography using an SP‐2330 column (0.32 mm ID, 30 m length) and a gas chromatograph equipped with dual autosamplers and dual flame ionization detectors. The SCD1 (Stearoyl‐CoA desaturase [Δ‐9‐desaturase]) index is the ratio of products (16:1n‐7 and 18:1n‐9) to precursor (16:0 and 18:0) fatty acids. The ELOVL6 (Fatty Acid Elongase 6) index is the ratio of products (18:0, 18:1n‐7 and 18:1n‐9) to precursor (16:0 and 16:1n‐7) fatty acids. The FADS1 (fatty acid desaturase 1) index was obtained from the ratio (20:4n6/20:3n6) and the FADS2 (fatty acid desaturase 2) index from the ratio (20:3n‐6)/(18:2n‐6).

### 2.8 Retrotranscription and real‐time PCR analysis

RNA was extracted from 50 mg of liver using the Trizol reagent in accordance with the manufacturer's instructions and 1 μg of RNA was converted to cDNA using M‐MLV Reverse Transcriptase, 100 ng random hexamers and 1 mM dNTPs in a final volume of 20 μL. Real‐time PCR was performed using Sybr Green primers (250 nmol) or Taqman primers and probes. Reactions were carried out using specific ABI Master Mixes (Applied Biosystem, Carlsbad, California) in a 7900HT Fast Real‐Time PCR System with a 384‐Well Block Module (Applied Biosystem, Carlsbad, California). Primers were designed using either Primer Express 2.0 or Primer Blast software (https://www.ncbi.nlm.nih.gov/tools/primer-blast/). (Sequences are available at http://tvp.mrl.ims.cam.ac.uk/primer-database-pagemax.) The geometrical average of 4 different genes (β2 microglobulin, β‐actin, 18S and 36B4) was used as an internal control, following an already described normalization method.^27^ Heat Maps were generated with the free software Multiple Experiment Viewer‐MeV‐ (http://mev.tm4.org/).

### 2.9 Western Blotting

Protein lysates from liver (50 mg) were run in NuPAGE 4%‐12% Bis‐Tris Protein Gels (Invitrogen, Carlsbad, California) and were electrotransferred to a nitrocellulose membrane using an iBlot Dry Blotting System (Thermofisher Scientific, Waltham, Massachusetts). All antibodies were from Cell Signaling Technology (Danvers, Massachusetts), with the exception of plin2/adrp and anti‐β‐actin, which were from Abcam (Cambridge, UK).

### 2.10 Statistical analysis

Two‐way or three‐way ANOVA was used for analysis of the interaction between genotype (G), diet (D) and treatment (T) for the fibrates intervention and between genotype (G), fasting (F) and/or diet (D) for the fasting cohort. IBM SPSS14 was used as statistical software.

## 3 RESULTS

### 3.1 P465L‐PPARγ mutant mice are hyperlipidaemic and hyperinsulinaemic

**Figure 1:**
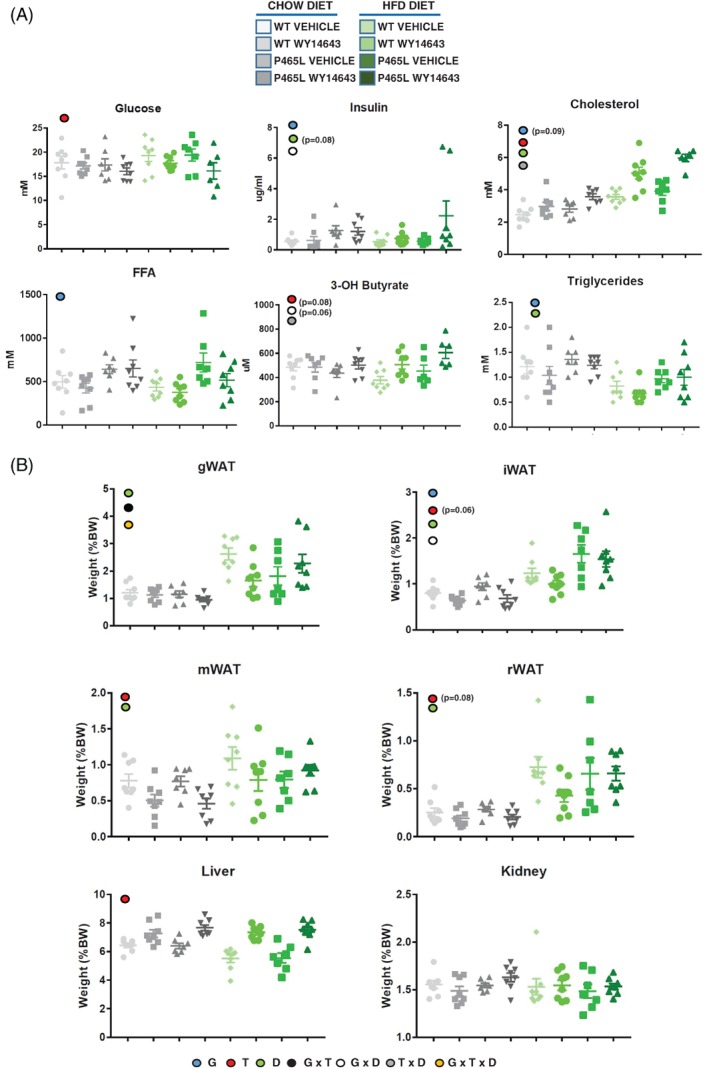
(A) Blood biochemistry and body composition (B) from P465L‐PPARγ mutant mice *vs* WT mice on a chow diet or an HFD, with or without WY14643 (ip:25 mg/kg). Graphs represent the average of 7‐8 mice per group ±SEM analysed by ANOVA (*P* < .05). Different coloured circles denote genotype effect (blue), treatment (red), diet (green), interactive effect of genotype × treatment (black), genotype × diet (white), diet × treatment (grey) and genotype × treatment × diet (orange)

P465L mutant mice showed higher levels of FFAs and TGs, and a tendency to increased cholesterol in serum, compared to WT mice (Figure 1A), independent of diet, recapitulating the hyperlipidaemia observed in human carriers of the P467L mutation. Both on a chow diet or after a short challenge with an HFD, P465L mice were normoglycaemic in the presence of hyperinsulinaemia (Figure 1A), suggesting that their insulin secretion was able to compensate for their peripheral insulin resistance; moreover, when challenged with an HFD for a period of 12 weeks, P465L mice became hyperglycaemic (Figure S1). The P465L mice also had elevated hepatic glycogen levels in the chow‐fed state (Figure S2A).

### 3.2 P465L‐PPARγ mutant mice are resistant to the pro‐lipolytic effect of WY14643 in adipose tissue

WY14643 treatment increased plasma cholesterol levels in both genotypes, as previously reported in other studies,^28^ and also increased levels of β‐hydroxybutyrate (BHB) in both genotypes (Figure 1A). The increase in BHB is compatible with fibrate‐mediated induction of hepatic β‐oxidation, a response that was slightly increased (ns) in P465L mice, particularly those on an HFD for 4 weeks. Interestingly, when fasted o/n, P465L mice showed significantly higher levels of BHB than WT mice (Figure S1), reflecting an increase in hepatic FFA delivery and β‐oxidation in P465L livers. At the organismal level, acute treatment with WY14643 induced hepatomegaly in both genotypes, coupled with a specific decrease in the fat mass of WT mice, especially in gWAT, but not in P465L mice on an HFD (Figure 1B and Figure S2). This difference in fat mass suggested that the P465L mutation may confer some degree of resistance against the pro‐lipolytic effect of fibrates in adipose tissue.^29^

### 3.3 P465L‐PPARγ mutation promotes fatty liver and alterations in lipoprotein metabolism

**Figure 2:**
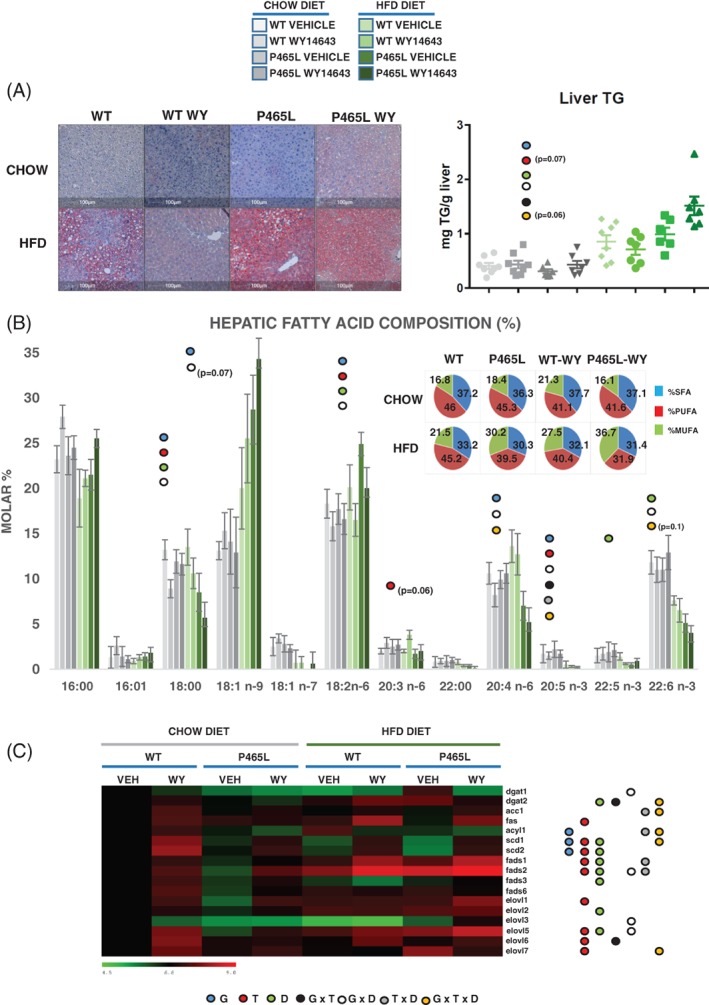
A, Oil red O‐stained sections (10×) and triglyceride composition of liver from P465L‐PPARγ mutant mice *vs* WT mice on a chow diet or an HFD, with or without WY14643 (ip:25 mg/kg). Graphs represent the average of 7‐8 mice per group ±SEM. B, Fatty acid composition in molar percentage of hepatic fatty acids from P465L‐PPARγ mutant mice *vs* WT mice on a chow diet or an HFD, with or without WY14643 (ip:25 mg/kg). C, Hepatic gene expression of candidate genes relevant in *de novo* lipogenesis and PUFA biosynthesis and analysed by ANOVA (*P* < .05). Different coloured circles denote genotype effect (blue), treatment (red), diet (green), interactive effect of genotype × treatment (black), genotype × diet (white), diet × treatment (grey) and genotype × treatment × diet (orange)

We have reported previously that P465L mice showed increased liver mass and hepatic accumulation of TG content when fed an HFD for 16 weeks or when backcrossed with the ob/ob mouse.^5^ In this new cohort of mice fed an HFD for only 28 days, the levels of hepatic TGs in P465L were already marginally higher (ns) than those in WT mice (Figure 2A). Unexpectedly, when mice on an HFD were treated with fibrates, we observed an increased hepatic fat content in P465L, but not in WT mice (Figure 2A). The analysis of macrovacuoles in HFD‐fed animals revealed a decrease in WT mice treated with fibrates, but not in P465L mice, evidencing a degree of resistance to the hypolipidaemic action of fibrates (data not shown). Similarly, P465L mice also presented more hepatic fat content after o/n fasting than their WT controls, further indicating that a large amount of the fat accumulated in the liver originated from adipose tissue (transient steatosis) (Figure S1). Overall, these data reinforced the hypothesis that PPARα activated mechanisms (eg, by response to fibrates/fasting) that regulate storage, oxidation and/or release of hepatic lipids were defective in P465L mice.

### 3.4 P465L‐PPARγ increases hepatic MUFA/PUFA ratio

We next analysed the chemical characteristics of hepatic fatty acid (Figure 2B). The liver of P465L mice on an HFD showed increased levels of monounsaturated fatty acids (MUFA). This was associated with an increased SCD1 index (Figure S2B) and active conversion of saturated (SFA) into MUFA, the preferred FA substrate for triglyceride storage. Despite the increase in the SCD1 index, *scd1* and *scd2* mRNA levels were downregulated, revealing a mismatch between enzymatic flux and gene regulation. The lower content of polyunsaturated fatty acids (PUFA) in the livers of HFD‐fed P465L mice was accounted for by reduced levels of 20:5 n‐3, 22:5 n‐3, 22:6 n‐3, 20:3 n‐6 and 20:4 n‐6, but not of 18:2 n‐6, the level of which was increased in P465L mice, indicating an impairment in the biosynthesis of PUFAs. It is of relevance that gene expression profiles did not reveal changes in the expression of elongases and fatty acid desaturases between genotypes (Figure 2C); however, the FADS1 index was downregulated in P465L livers. Interestingly, the FADS2 index was significantly increased in WT mice treated with WY14643, but to a lesser extent than in P465L mice (Figure S2B). Interestingly, the expression of *fads3*, a putative desaturase associated with changes in PUFA levels,^30^ was downregulated in P465L livers (Figure 2C), suggesting that FADS3 may be a new fatty acid desaturase under the transcriptional regulation of PPARγ. Gene expression profiling of genes and analysis of proteins involved in *de novo* lipogenesis evidenced an increase in their expression/levels in response to fibrates (Figure 2C and Figure S4A), consistent with the concept that fibrates promote, not only catabolic, but also anabolic lipid pathways.

### 3.5 P465L‐PPARγ reduces serum VLDL levels in HFD fed mice

**Figure 3:**
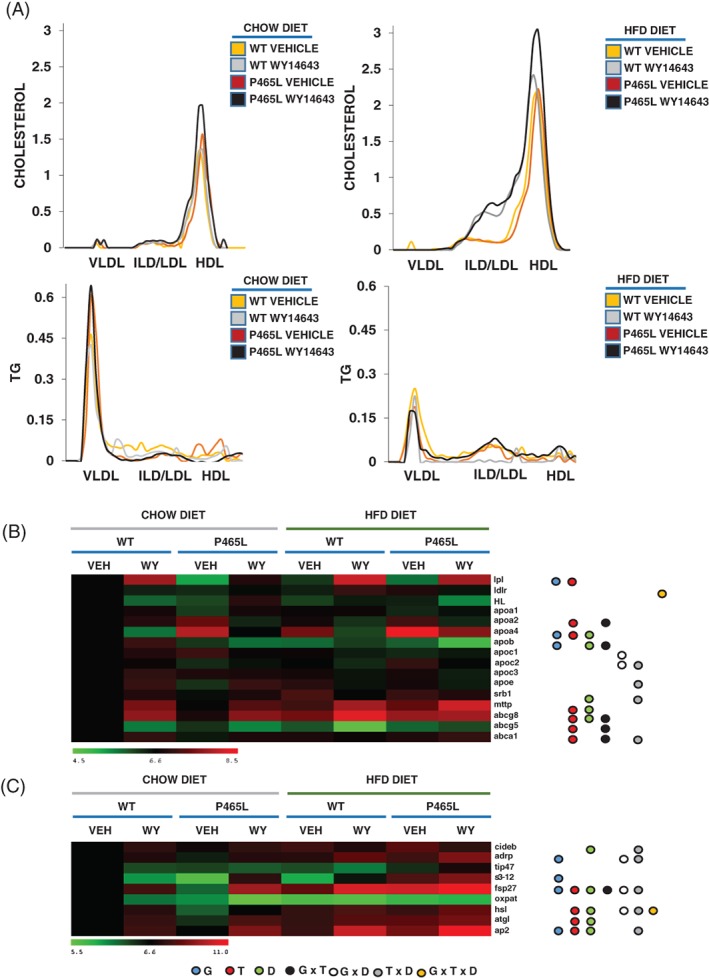
A, Lipoprotein cholesterol and TG distribution were determined in plasma from non‐fasted P465L‐PPARγ mutant mice *vs* WT mice on a chow diet or an HFD, with or without WY14643 (ip:25 mg/kg). Approximate elution volumes for particles in the size ranges of VLDL, LDL and HDL are indicated. B, Hepatic gene expression of candidate genes relevant in lipoprotein metabolism and C, lipid droplet proteins is shown as log2 conversions of average gene expression data relative to controls (log2 100 = 6.6). Magnitude >6.6 and <6.6 denotes up‐ and downregulation, respectively, compared with WT controls on a chow diet, analysed by ANOVA (*P* < .05). Different coloured circles denote genotype effect (blue), treatment (red), diet (green), interactive effect of genotype × treatment (black), genotype × diet (white), diet × treatment (grey) and genotype × treatment × diet (orange)

**Figure 4:**
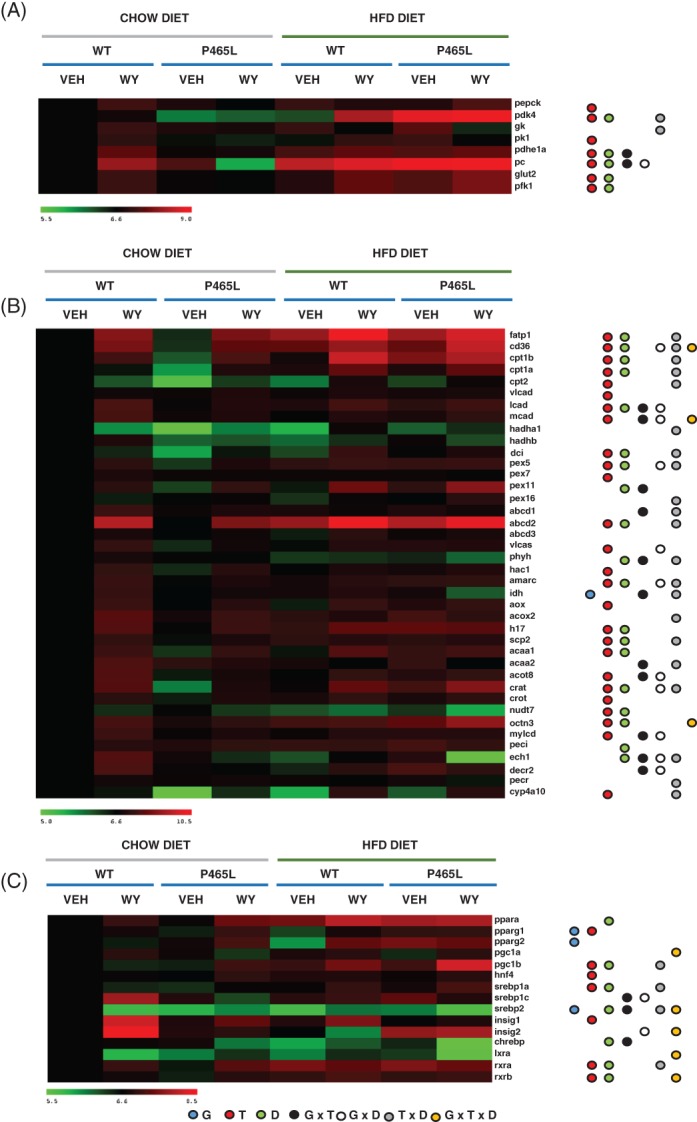
Hepatic gene expression of candidate genes relevant in A, glucose metabolism, B, fatty acid uptake and mitochondrial/peroxisomal fatty acid oxidation programmes, and C, nuclear transcription factors is shown as log2 conversions of average gene expression data relative to controls (log2 100 = 6.6). Magnitude >6.6 and <6.6 denotes up‐ and downregulation, respectively, compared with WT controls on a chow diet, analysed by ANOVA (*P* < .05). Different coloured circles denote genotype effect (blue), treatment (red), diet (green), interactive effect of genotype × treatment (black), genotype × diet (white), diet × treatment (grey) and genotype × treatment × diet (orange)

We next investigated whether changes in the lipoprotein lipid composition in P465L mice mirrored the increased fat accumulation in liver, as well as the identified blunted functional effect of fibrates (Figure 3A,B). It has been reported previously that the hypolipidaemic effects of fibrates are mostly the result of enhanced catabolism of TG‐rich particles (increased lipoprotein lipase, LPL and decreased Apolipoprotein CIII, apoCIII) and of a decrease in Apolipoprotein B (ApoB) and VLDL production. Analysis of VLDL in chow‐fed conditions revealed that plasma levels of VLDL‐TG in P465L mice were greater than in WT mice. *A priori,* these increased levels of VLDL may have been accounted for by the hyperinsulinaemia of the P465L mice, leading to increased hepatic influx of FFA, accumulation of TG and increased VLDL‐TG secretion. However, what was not expected was that, when on an HFD, P465L mice showed a paradoxical decrease in VLDL‐TG levels in plasma compared to WT controls. Administration of WY14643 did not change VLDL‐TG levels in mice of any genotype on a chow diet, but administration of WY14643 to WT mice on an HFD slightly decreased VLDL‐TG, in line with the effects of fibrates in VLDL secretion (Figure 3A). *ApocIII* expression showed no genotype‐dependent differences and, as previously mentioned, P465L mice showed a reduction in plasma VLDL‐TG levels, despite high fatty acid levels in serum. Of potential pathogenic relevance in fatty liver development, P465L livers showed increased expression of lipid droplet proteins such as adipophilin/perilipin 2 (*adrp/plin2*) (Figure 3C and Figure S4A), fat‐specific protein 27/cell death‐inducing DFFA‐like effector C *(fsp27/cidec)* and s3‐12/perilipin 4 (*s3‐12/plin4),* as well as fatty acid‐binding protein 4/adipocyte protein 2 (*fabp4/ap2)* (Figure 3C and Figure 4A).

Gene expression analysis, in chow‐fed mice, of genes involved in transport of lipids confirmed that mRNA levels of hepatic (Figure 3B) and adipose LPL (Figure S3A) and of fatty acid transporters *cd36* and *fatp1* in skeletal muscle (Figure S3B) were decreased in P465L mice, in agreement with the impaired peripheral metabolism of VLDL‐TG in P465L mutants. Of note, *apoIV* gene expression, which has been associated previously with hepatic steatosis and with increased secretion of larger TG‐enriched apoB‐containing VLDL,^31^ was increased in P465L livers, independent of the nutritional and pharmacological challenge. We also observed a genotype‐dependent decrease in levels of *apob* and minor differences in *mttp* (microsomal triglyceride transfer protein).

We subsequently analysed the effect of P465L on HDL lipoproteins. Unlike in humans, where fibrates increase HDL‐C levels and *apoa1* expression,^32^ fibrates have been reported to have no effect on, and do not even decrease levels of HDL and *apoa1* expression in non‐transgenic wild‐type mice.^33^, ^34^ Surprisingly, in P465L mice fed both a chow diet and an HFD, fibrates increased HDL levels in comparison to WT mice. This finding indicates that the P465L‐PPARγ mutation interferes, directly or indirectly, with the normal response to fibrates in HDL metabolism.

Moreover, we observed an increased *apoa2/apoa1* mRNA ratio in P465L mice (Figure 3B). Of note, increased levels of *apoa2* have been associated with increased pro‐oxidative and pro‐inflammatory responses, with alterations in the rate of HDL metabolization and with increased atherogenic risk.^35^ Thus, this evidence indicates that the changes observed in P465L mutants result in a deleterious effect on HDL metabolism.

WY14643 also increased cholesterol enriched IDL/LDLP465L‐PPAR‐C levels in both genotypes. A similar effect has been reported previously for fenofibrate in mice fed a high fat/high sucrose diet^36^ and in patients with severe dyslipidaemia.^37^ We observed that, in mice on an HFD, treatment with WY14643 reduced the triglyceride‐enriched IDL/LDL‐TG in WT mice, but not in P465L mice (Figure 2B). This is in agreement with a decrease in the metabolization of TG from the IDL/LDL fraction, probably mediated by hepatic or peripheral lipases in P465L mice, where both levels of hepatic lipase (HL) and low‐density lipoprotein receptor (LDLR) are reduced as compared to WT mice.

Globally considered, these findings indicate that the increase in triglyceride‐enriched VLDL‐TG plasma levels in chow‐fed P465L mice was the result of the increased flux of FFA into the liver, coupled with increased VLDL secretion and reduced peripheral/hepatic catabolism. Our data also show a dietary‐related differential response that is characterized by increased VLDL levels linked to an increased flux of lipids into the liver under chow‐fed conditions in P465L mutant mice. This contrasted with the HFD phenotype defined by lower VLDL levels and increased hepatic steatosis. These paradoxical responses suggest that the P465L mutant exhibits decreased metabolic flexibility related to hepatic lipid handling, which becomes evident in the context of specific lipid‐related nutritional and pharmacological challenges.

### 3.6 Hepatic mitochondrial and peroxisomal FAO genes are downregulated in HFD fed P465L mice

Fibrates promote a metabolic switch that favours the use of fatty acid as energetic substrates. Fibrates are known to activate the pyruvate dehydrogenase kinases (PDK2 and PDK4), which inactivate pyruvate dehydrogenase and enhance the utilization of serum fatty acids and triglycerides.^38^ P465L livers showed reduced hepatic expression of *pdk4* and a blunted response to WY14643 in comparison to WT livers (Figure 4A). Thus, the P465L mutation prevents the induction of fatty acid oxidation (FAO) by WY14643. The question was whether P465L‐PPARγ induced dysfunction in the mitochondrial and/or peroxisomal FAO programmes that could contribute to the development of fatty liver and liver damage in P465L mice, as shown in other models.^39^, ^40^

P465L mutant mice showed reduced expression of mFAO and pFAO genes in chow‐fed non‐fibrate‐treated mice *vs* WT counterparts (Figure 4B). These data may be interpreted as either P465L‐PPARγ preventing basal expression of genes regulated by PPARα or, alternatively, physiological expression of PPARγ itself possibly directly impairing expression of these genes. The latter is unlikely, given that hepatic expression of PPARγ under normal physiological conditions (ie, chow diet) is limited.

There is substantial literature showing a pro‐oxidative role for PPARα.^41^, ^42^ In line with this, WY14643 increased the expression of most of the genes associated with mFAO and pFAO programmes in both genotypes. However, we identified a set of genes for mFAO and pFAO programmes that failed to be upregulated in P465L livers in response to WY14643. Specifically, the ATP‐binding cassette subfamily D member 1 (*abcd1),* phytanoyl‐CoA 2 hydroxylase *(phyh),* isocitrate dehydrogenase *(idh),* enoyl‐CoA hydratase 1 *(ech1),* 2,4‐dienoyl‐CoA reductase 2 *(decr2),* failed to be induced by fibrates in P465L liver, providing evidence of selective resistance to the transcriptional response to fibrates (Figure 4B). We also identified genes that responded differently in P465L livers *vs* WT livers when fed chow or HFD diets (Long chain acyl‐CoA dehydrogenase, *lcad*; medium chain Acyl‐CoA dehydrogenase *mcad;* very long chain acyl‐CoA synthase *vlcas;* alpha methyl‐acyl‐CoA racemase *amacr;* malonyl‐CoA decarboxylase*, mylcd*). This suggested an interaction effect of the genotype with both fibrate treatment and dietary challenges.

When assessing the expression of FAO genes in mice fasted overnight we did not observe strong changes associated with the P465L mutation apart from abcd1, scp2 (GxF interactive effect) (Figure S4).

Finally, we also evaluated the expression of transcription factors other than pparα and pparγ that are involved in the transcriptional activation of multiple lipid‐related genes (Figure 4C) and whose expression could also be dysregulated by the activation of pparα (fibrates) and the P465L mutation. In this regard, fibrates and P465L affected the expression of *srebp1*, *hnf4*, *rxr* (Figure 4C). Interestingly, the expression of pparγ at gene and protein levels (Figure 4C and Figure S4A) was upregulated in P465L mutant livers, increasing the pool of the WT and also the mutant P465L‐ PPARγ, and thus competing with pparα in response to fibrates when both transcription factors are upregulated (Figure 4C and Figure S4A).

### 3.7 P465L‐PPARγ interferes with the transrepression capacity of PPARα

**Figure 5:**
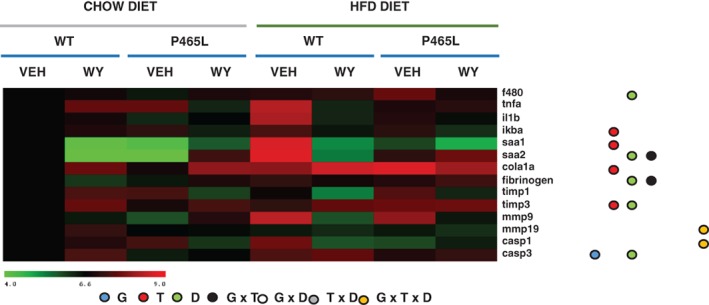
Hepatic gene expression of candidate genes relevant in inflammation and fibrosis is shown as log2 conversions of average gene expression data relative to controls (log2 100 = 6.6). Magnitude >6.6 and <6.6 denotes up‐ and downregulation, respectively, compared with WT controls on a chow diet, analysed by ANOVA (*P* < .05). Different coloured circles denote genotype effect (blue), treatment (red), diet (green), interactive effect of genotype × treatment (black), genotype × diet (white), diet × treatment (grey) and genotype × treatment × diet (orange)

Fibrates also exert anti‐fibrotic and anti‐inflammatory effects through transrepressive mechanisms.^43^ We observed that, in HFD WT mice, administration of WY14643 reduces the expression of target genes such as *serum amyloid A* (*saa1* and *saa2)* and *fibrinogen* (Figure 5). Interestingly, this effect was not observed in P465L mice, suggesting that this mutation also prevented the transrepression activity of PPARα.

## 4 DISCUSSION

This work follows previous research from our group and others concerning the P465L mouse, a humanized model for the dominant negative mutant P467L‐PPARγ that resembles the phenotype observed in patients and is characterized by a partial lipodystrophy, insulin resistance, hypertension and fatty liver.

Here, we provide evidence that the fatty liver observed in the P465L‐PPARγ knock‐in mouse involves a selective impairment in the transcriptional activation of PPARα. This is supported by *in vivo* data showing that P465L mice developed fatty liver on an HFD and were resistant to treatment with WY14643, a fibrate that acts as a PPARα activator, determining a phenotype highly reminiscent of the resistance to fibrates previously observed in PPARα KO mice.^44^ We have shown that, in response to WY14643, the size of the fat depots is decreased in WT mice, whereas P465L adipose tissue remains unaffected. Similarly, whereas WY14643‐treated HFD WT mice showed a reduction in hepatic TG content, the P465L did not, and, additionally, we observed exacerbated hepatic accumulation of TG during the fed/fasting transition in P465L mice fed a chow diet or an HFD. These responses are supportive of a partial resistance to the effects of PPARα activation in P465L mice.

We have learned that the PPARγ‐P465L mutant interferes with the lipoprotein profile and its regulation by fibrates. We observed a shift in the lipoprotein fingerprint between chow‐ and HFD‐fed P465L mice. When on a chow diet, P465L mice had increased levels of VLDL compared to WT mice, an observation consistent with an increased flux in fatty acids originating in the leak from dysfunctional adipose tissue and associated hyperinsulinaemia. However, when fed an HFD for 28 days, the pattern of VLDLs was reversed, with decreased levels of VLDL coupled with increased expression of hepatic lipid droplet proteins in the P465L‐PPARγ mutant livers. Whether this increase in lipid droplet protein expression in mutant livers is directly mediated by a disordered transcriptional programme of PPARγ, or is merely a secondary perturbation, is unclear. It is well known that PPARγ and PPARα are both transcriptional regulators of *adrp/plin2* and *fsp27/cidec* 45 and that the overexpression of *fsp27/cidec and adrp/plin2* prevents the access of lipases to the core of the lipid droplet, thus impairing the hydrolysis of TG.^46^, ^47^ Therefore, the increased expression of lipid droplet proteins may be a relevant pathogenic factor for hepatic steatosis,^48^ contributing to the accumulation of lipids in P465L mice. Of note, *fsp27/cidec* expression is further increased in P465L livers with fibrates, which may contribute to the increase in hepatic TG levels in HFD‐fed P465L mice in comparison to WT mice. This apparent paradox of PPARα simultaneously promoting the expression of lipid droplet proteins that limit the mobilization of lipid from the liver, together with PPARα promoting lipid oxidation, suggests the relevance of PPARα for the activation of genetic programmes that aim to reduce the accumulation of potential toxic lipid species by a double strategy that involves either diverting lipids to a safe storage or metabolizing them. This duality of functions may explain the apparent controversially disparate results observed with the use of fibrates to treat fatty liver in several rodent models,^49^, ^50^ particularly when one of these two programmes may prevail over the other.

It is assumed that the association between lipodystrophy and NAFLD is a direct consequence of the failure of adipose tissue to expand and the consequent spillage of excessive lipids into the liver. Here, we provide evidence that the fatty liver of the P465L lipodystrophic mice exhibits, not only quantitative, but also qualitative changes (increased MUFA/PUFA ratio) in the accumulated lipids. These data are consistent with changes in hepatic lipid biosynthetic pathways that are characteristic of “classical models” of NAFLD and indicate that these qualitative changes in the fatty acid pool of P465L livers may reflect a common pathogenic signature of NAFLD, rather than a specific fingerprint driven by the presence of the PPARγ mutation.

Our expression profiling revealed selective impairment of PPARα preferentially regulated genes in P465L mice on a chow diet and treated only with vehicle. This indicates that, even when PPARα ligands are present at low physiological levels, the presence of the P465L mutation is enough to dysregulate the expression of those PPARα genes at a transcriptional level. The P465L mice on a chow diet also showed impaired expression of genes controlling mFAO and pFAO, well known targets of PPARα in mice. These effects were partially masked after treatment with WY14643 and/or an HFD. Additionally, we identified genes in the P465L livers that remained pathologically unresponsive to treatment with fibrates in comparison to WT livers, providing compelling evidence of resistance to the ligand‐dependent activation of PPARα when P465L‐PPARγ is present. Interestingly, genotype‐driven differences were reduced when mice were fasted overnight (GxF interactive effect), indicating that the effect of the P465L mutation on PPARα function may become pathophysiologically relevant only at basal levels, when the expression/activity of PPARα is below a particular threshold or, alternatively, that it may be overcome by the hormonal adaptation taking place in response to starvation.

Despite the fact that our experimental model of treatment with HFD/WY14643 was not specifically designed to induce severe liver damage, we found that the expression of genes involved in fibrosis and known to be downregulated by PPARα activation were altered in P465L livers, suggesting that transrepression activity of PPARα was impaired also by the presence of the P465L‐PPARγ mutant.

Globally considered, our data indicate that the pathogenesis of the fatty liver observed in P465L mice is more complex than being simply the result of the failure of the adipose tissue, and that it involves the combination of several pathogenic factors at the hepatic level, including the uncoupling of lipid storage in lipid droplets from the assembly, and transport and secretion of VLDL, associated with impaired hepatic FAO in a pathological context where peripheral uptake of lipids is likely to be compromised.

In summary, we have shown that P465L mice develop hepatic steatosis that is associated with increased lipid trapping and impaired VLDL secretion with HFD, resulting in qualitative changes in the hepatic fatty acids that recapitulate the fingerprint of common NAFLD models. We also provide biochemical data, hepatic lipid content and evidence of impaired expression of a number of well established PPARα target genes in P465L livers that supports the conclusion that P465L confers partial resistance to the hypolipidaemic action of fibrates. Moreover, our results show that the fatty liver phenotype observed in P465L mutant mice is not only the consequence of dysfunctional adipose tissue, but also involves defective liver metabolism. Despite the current lack of trials addressing the efficacy of fibrates in patients with partial lipodystrophy, our results raise concerns regarding the potential value of fibrates for the management of hypertriglyceridaemia/NAFLD in carriers of the dominant negative P467L‐PPARγ mutation. Whether our findings are transferable to the overall FPLD3 spectrum of diseases is currently unknown. However, there are reports of FPLD3 patients, characterised by recurrent hypertriglyceridaemia despite treatment with fibrates.^20^, ^21^, ^22^, ^23^

Finally, our data also indicate that the specific repertoire of PPARs present under specific metabolic conditions is important, as it may modulate the fine balance between transcription factors with metabolically opposed functions.

### Conflict of interest

The authors declare that they have no conflict of interest.

### Author contributions

S. R. C. and S. C. conceived the original hypothesis, designed and performed experiments *in vivo/ex vivo* and wrote the manuscript. G. B. C. performed the fatty acid composition analysis, and discussed and edited the manuscript. X. P. performed the lipoprotein profiling, and discussed and edited the manuscript. R. A., J. R., M. C., R. D. and M. B. contributed to *ex vivo* profiling, and discussed and edited the manuscript. S. G. conceived the original hypothesis, designed experiments and wrote the manuscript. A. V. P. conceived the original hypothesis, designed experiments and wrote the manuscript, and is the guarantor of this work. All authors approved its publication.

## Supporting information


**FIGURE S1** Blood biochemistry from P465L pparγ mutant mice vs. WT mice fed chow or HFD for 3M in the fed and fasted state. Graphs represent the average of 5‐8 mice per group ±SEM analysed by ANOVA (*P* < .05). Different colour circles denote Genotype effect (blue), fasting (red), diet (green), interactive effect genotype × fasting (black), genotype × diet (white), diet × fasting (grey) and genotype × fasting × diet (orange)

**FIGURE S2** A, Fat percentage, lean mass, bone mineral density (BMD) and Hepatic glycogen levels in P465L pparγ mutant mice vs. WT mice fed chow or HFD with or without WY14643 (ip:25 mg/kg). B, Calculated SCD1, ELOVL6 and FADS1‐2 ratio from data shown in Figure 2B. Graphs represent the average of 5‐8 (B) mice per group ±SEM and analysed by ANOVA (*P* < .05). Different colour circles denote Genotype effect (blue), treatment (red), diet (green), interactive effect genotype × treatment (black), genotype × diet (white), diet × treatment (grey) and genotype × treatment × diet (orange)

**FIGURE S3** A, Gene expression in gonadal adipose tissue (A) and skeletal muscle (B) is shown as log2 conversions of average gene expression data relative to control (log2 100 = 6.6). Magnitude >6.6 and <6.6 denotes up‐ and downregulation, respectively, compared with WT, chow fed controls. B, Hepatic levels of glycogen. Graphs represent the average of 7‐8 mice per group ±SEM and analysed by ANOVA (*P* < .05). Different colour circles denote Genotype effect (blue), treatment (red), diet (green), interactive effect genotype × treatment (black), genotype × diet (white), diet × treatment (grey) and genotype × treatment × diet (orange)

**FIGURE S4** A, Expression of proteins involved in lipid droplet scaffolding, de novo lipogenesis and the transcription factors pparγ and pparα. B, Expression of genes relevant for liver metabolism from P465L pparγ mutant mice vs. WT mice fed HFD for 12w in the fed and fasted state is shown as log2 conversions of average gene expression data relative to control (log2 100 = 6.6). Magnitude >6.6 and <6.6 denotes up‐ and downregulation, respectively, compared with WT, HFD fed controls. Graphs represent the average of 6‐8 mice per group ±SEM and analysed by ANOVA (*P* < .05). Different colour circles denote Genotype effect (blue), fasting (red), and interactive effect genotype × fasting (black)
