# Supplementary material for: P465L‐PPARγ mutation confers partial resistance to the hypolipidaemic action of fibrates
Source: Diabetes Obes Metab. 2018 Jun 27;20(10):2339–50. doi: 10.1111/dom.13370 (PMC6589924; doi:10.1111/dom.13370)
Supplement: Supplementary file 1 — FIGURE S1 Blood biochemistry from P465L pparγ mutant mice vs. WT mice fed chow or HFD for 3M in the fed and fasted state. Graphs represent the average of 5‐8 mice per group ±SEM analysed by ANOVA (P < .05). Different colour circles denote Genotype effect (blue), fasting (red), diet (green), interactive effect genotype × fasting (black), genotype × diet (white), diet × fasting (grey) and genotype × fasting × diet (orange) [file DOM-20-2339-s001.pptx]

## Slide 1
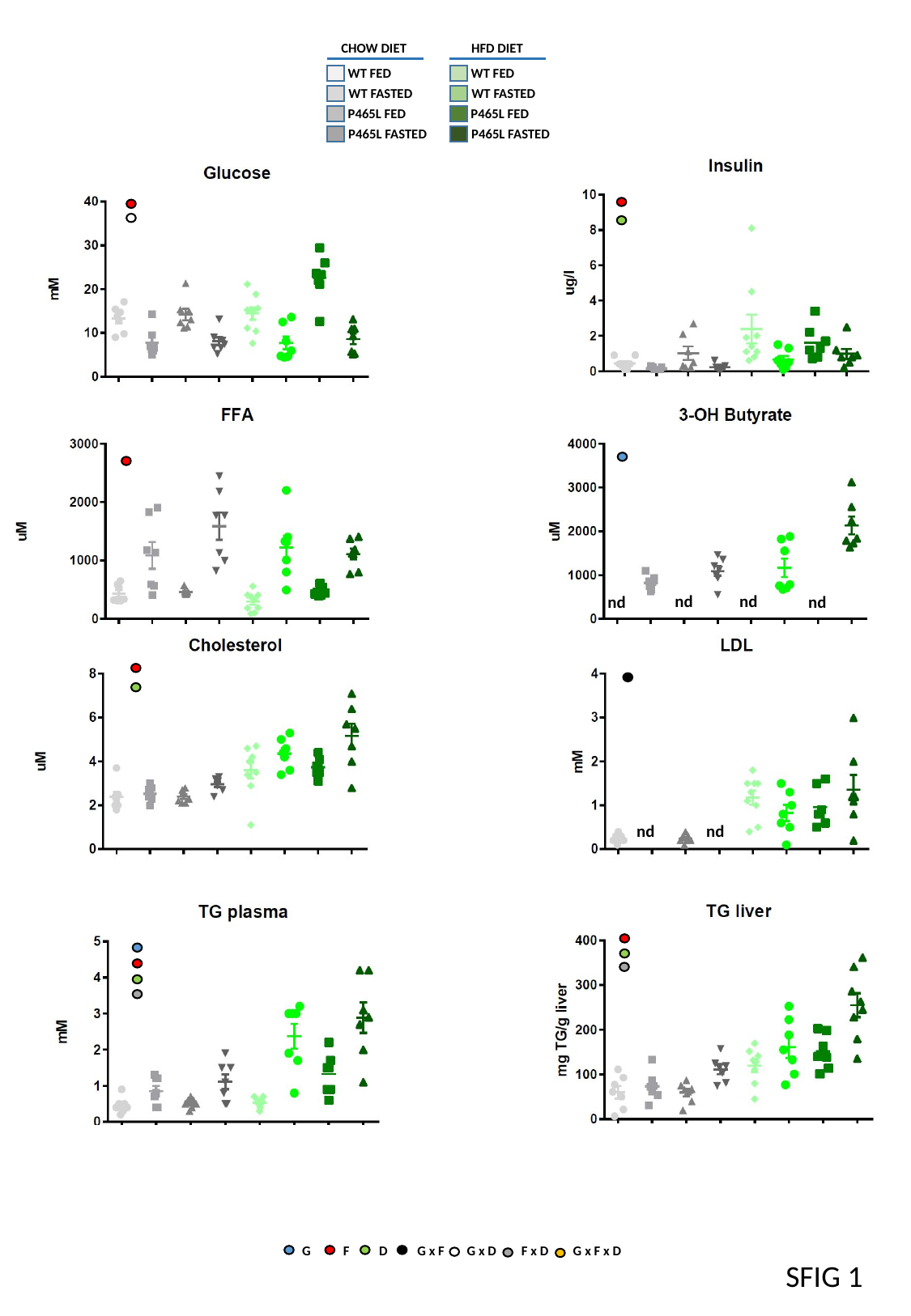

CHOW DIET
HFD DIET
WT FED
WT FED
WT FASTED
WT FASTED
P465L FED
P465L FED
P465L FASTED
P465L FASTED
nd
nd
nd
nd
nd
nd
G
F
D
G x F
G x D
F x D
G x F x D
SFIG 1
