# Supplementary material for: P465L‐PPARγ mutation confers partial resistance to the hypolipidaemic action of fibrates
Source: Diabetes Obes Metab. 2018 Jun 27;20(10):2339–50. doi: 10.1111/dom.13370 (PMC6589924; doi:10.1111/dom.13370)
Supplement: Supplementary file 2 — FIGURE S2 A, Fat percentage, lean mass, bone mineral density (BMD) and Hepatic glycogen levels in P465L pparγ mutant mice vs. WT mice fed chow or HFD with or without WY14643 (ip:25 mg/kg). B, Calculated SCD1, ELOVL6 and FADS1‐2 ratio from data shown in Figure 2B. Graphs represent the average of 5‐8 (B) mice per group ±SEM and analysed by ANOVA (P < .05). Different colour circles denote Genotype effect (blue), treatment (red), diet (green), interactive effect genotype × treatment (black), genotype × diet (white), diet × treatment (grey) and genotype × treatment × diet (orange) [file DOM-20-2339-s002.pptx]

## Slide 1
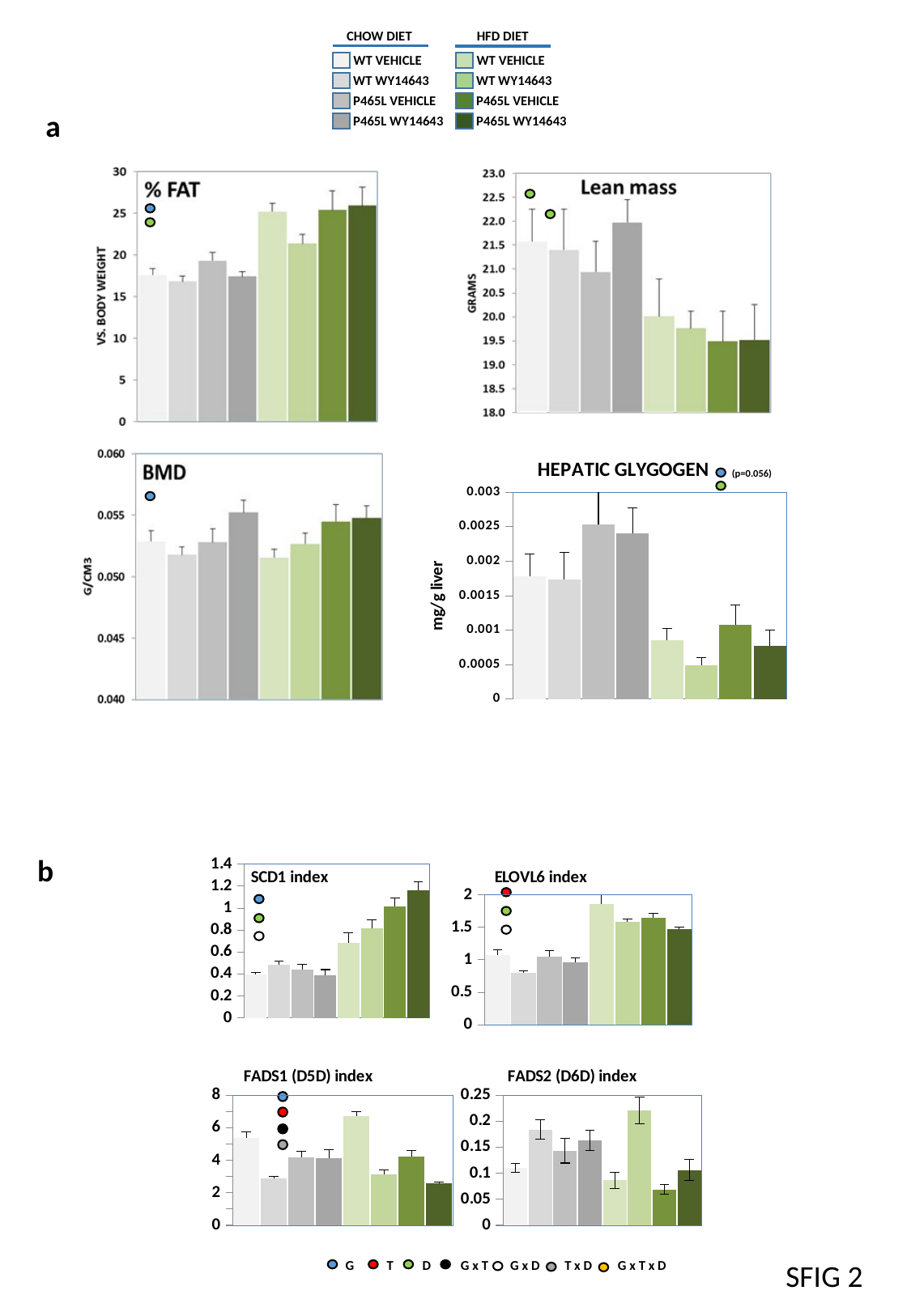

CHOW DIET
HFD DIET
WT VEHICLE
WT VEHICLE
WT WY14643
WT WY14643
P465L VEHICLE
P465L VEHICLE
P465L WY14643
P465L WY14643
a
### Chart: HEPATIC GLYGOGEN
| Category | |
|---|---|
| WT | 0.001785259345050681 |
| MUT | 0.001741158743531829 |
| WTF | 0.0025284409548853477 |
| MUTF | 0.00240452583371234 |
| WT | 0.0008506409893771305 |
| MUT | 0.0004905067579798249 |
| WTF | 0.0010816449726867025 |
| MUTF | 0.0007728745271669201 |
 (p=0.056)
b
### Chart: SCD1 index
| Category | |
|---|---|
### Chart: ELOVL6 index
| Category | |
|---|---|
### Chart: FADS1 (D5D) index
| Category | |
|---|---|
### Chart: FADS2 (D6D) index
| Category | |
|---|---|
G
T
D
G x T
G x D
T x D
G x T x D
SFIG 2
