# Supplementary material for: P465L‐PPARγ mutation confers partial resistance to the hypolipidaemic action of fibrates
Source: Diabetes Obes Metab. 2018 Jun 27;20(10):2339–50. doi: 10.1111/dom.13370 (PMC6589924; doi:10.1111/dom.13370)
Supplement: Supplementary file 3 — FIGURE S3 A, Gene expression in gonadal adipose tissue (A) and skeletal muscle (B) is shown as log2 conversions of average gene expression data relative to control (log2 100 = 6.6). Magnitude >6.6 and <6.6 denotes up‐ and downregulation, respectively, compared with WT, chow fed controls. B, Hepatic levels of glycogen. Graphs represent the average of 7‐8 mice per group ±SEM and analysed by ANOVA (P < .05). Different colour circles denote Genotype effect (blue), treatment (red), diet (green), interactive effect genotype × treatment (black), genotype × diet (white), diet × treatment (grey) and genotype × treatment × diet (orange) [file DOM-20-2339-s003.pptx]

## Slide 1
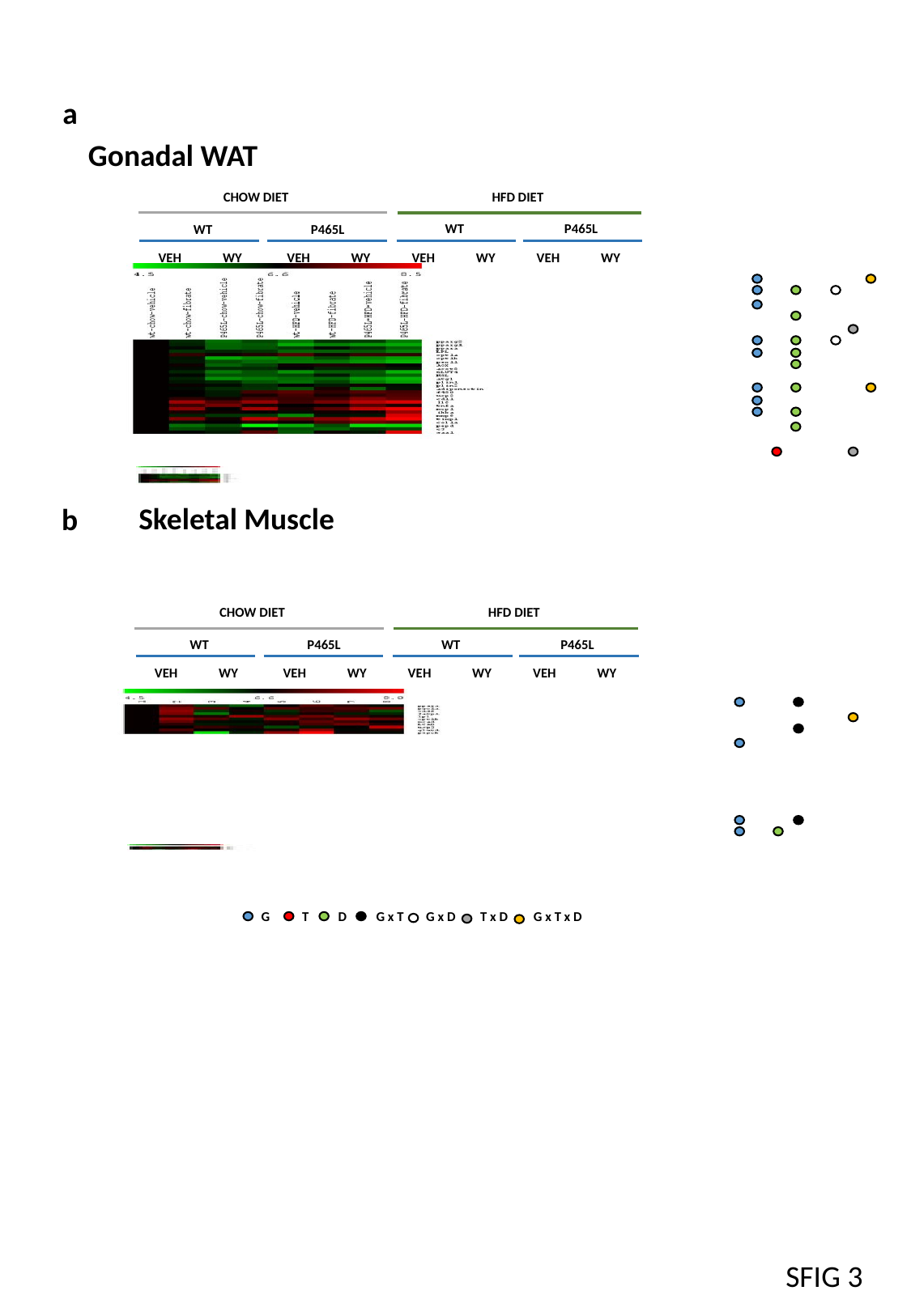

a
Gonadal WAT
CHOW DIET
HFD DIET
P465L
WT
P465L
WT
VEH
WY
VEH
WY
VEH
WY
VEH
WY
Skeletal Muscle
b
CHOW DIET
HFD DIET
P465L
WT
P465L
WT
VEH
WY
VEH
WY
VEH
WY
VEH
WY
G
T
D
G x T
G x D
T x D
G x T x D
SFIG 3
