# Supplementary material for: P465L‐PPARγ mutation confers partial resistance to the hypolipidaemic action of fibrates
Source: Diabetes Obes Metab. 2018 Jun 27;20(10):2339–50. doi: 10.1111/dom.13370 (PMC6589924; doi:10.1111/dom.13370)
Supplement: Supplementary file 4 — FIGURE S4 A, Expression of proteins involved in lipid droplet scaffolding, de novo lipogenesis and the transcription factors pparγ and pparα. B, Expression of genes relevant for liver metabolism from P465L pparγ mutant mice vs. WT mice fed HFD for 12w in the fed and fasted state is shown as log2 conversions of average gene expression data relative to control (log2 100 = 6.6). Magnitude >6.6 and <6.6 denotes up‐ and downregulation, respectively, compared with WT, HFD fed controls. Graphs represent the average of 6‐8 mice per group ±SEM and analysed by ANOVA (P < .05). Different colour circles denote Genotype effect (blue), fasting (red), and interactive effect genotype × fasting (black) [file DOM-20-2339-s004.pptx]

## Slide 1
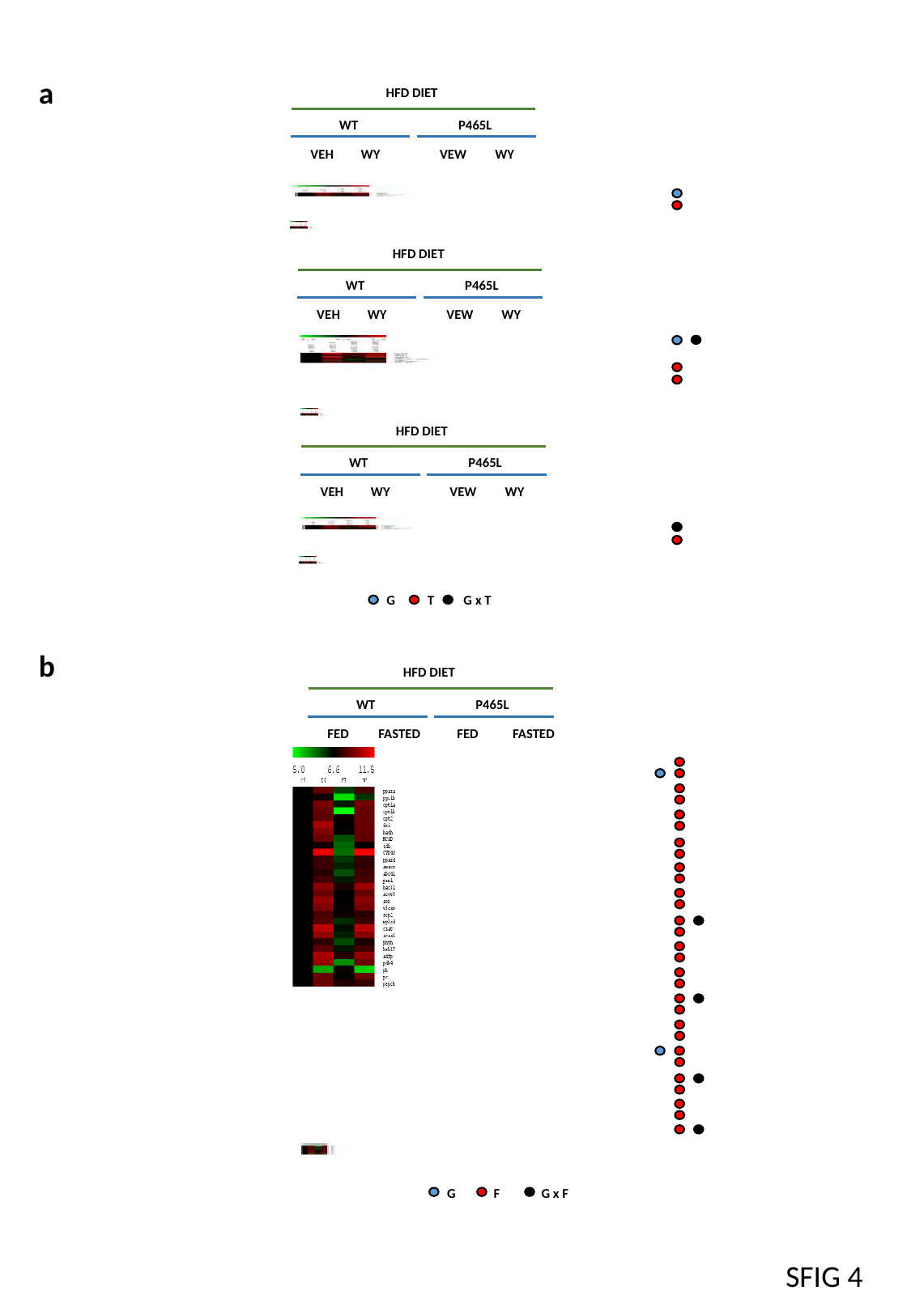

a
HFD DIET
P465L
WT
VEH
WY
VEW
WY
HFD DIET
P465L
WT
VEH
WY
VEW
WY
HFD DIET
P465L
WT
VEH
WY
VEW
WY
G x T
G
T
b
HFD DIET
P465L
WT
FED
FASTED
FED
FASTED
G
F
G x F
SFIG 4
